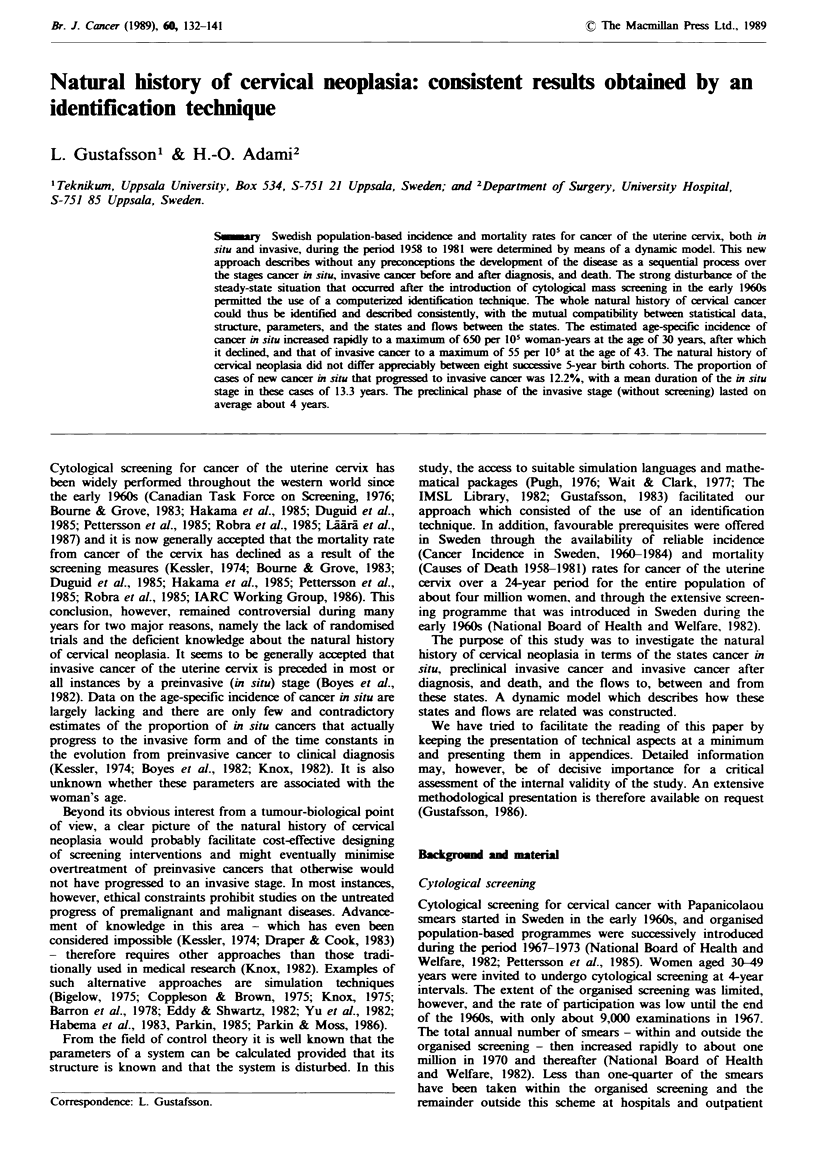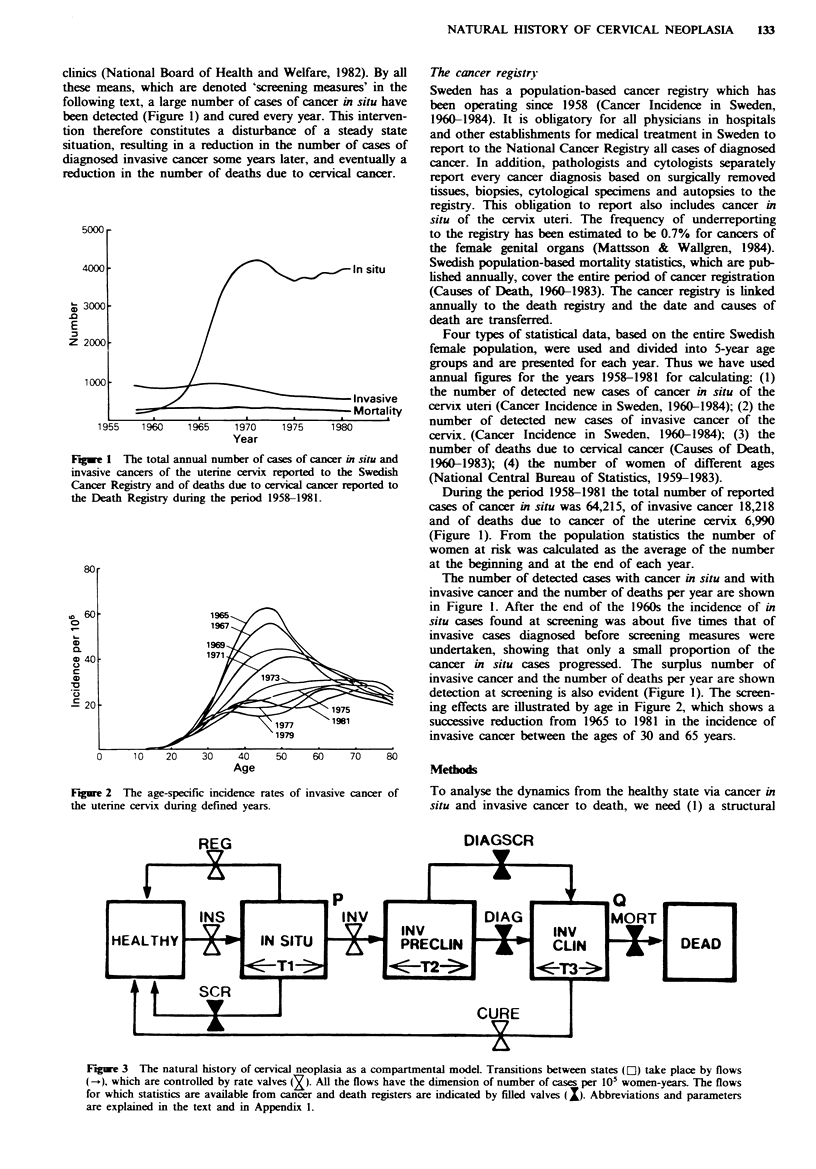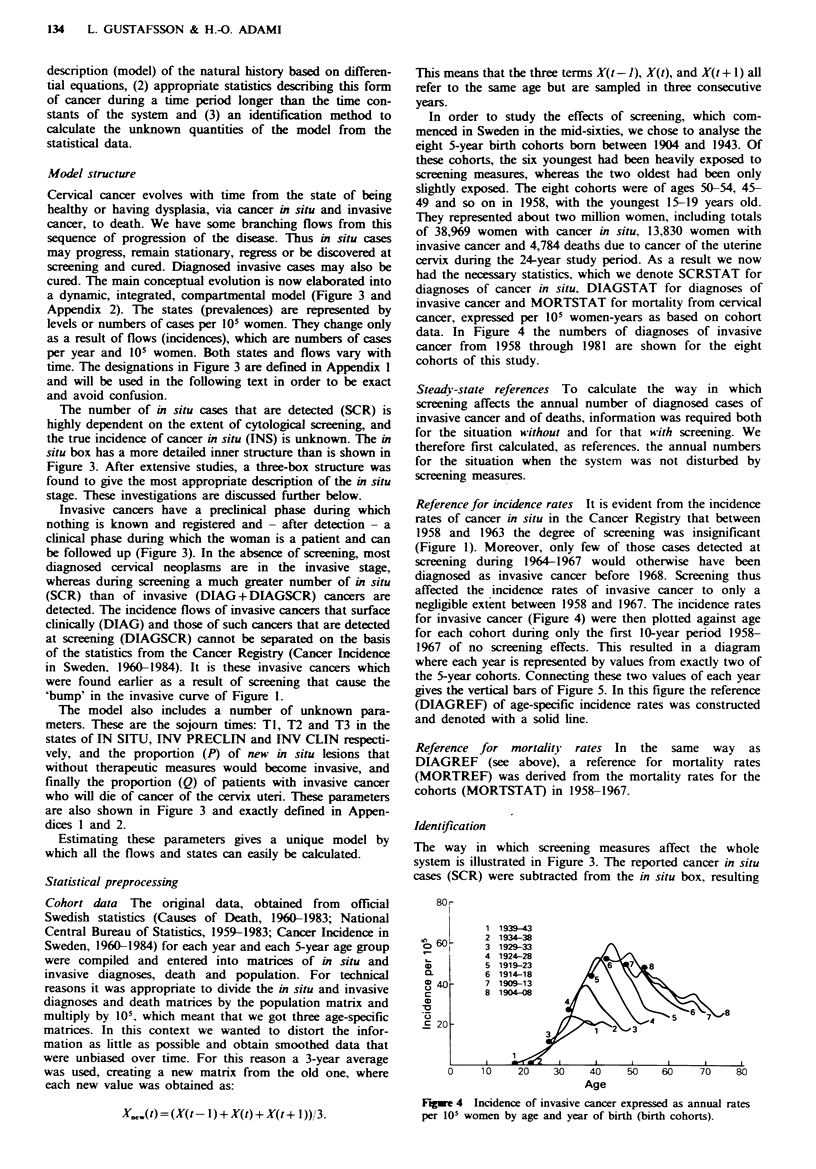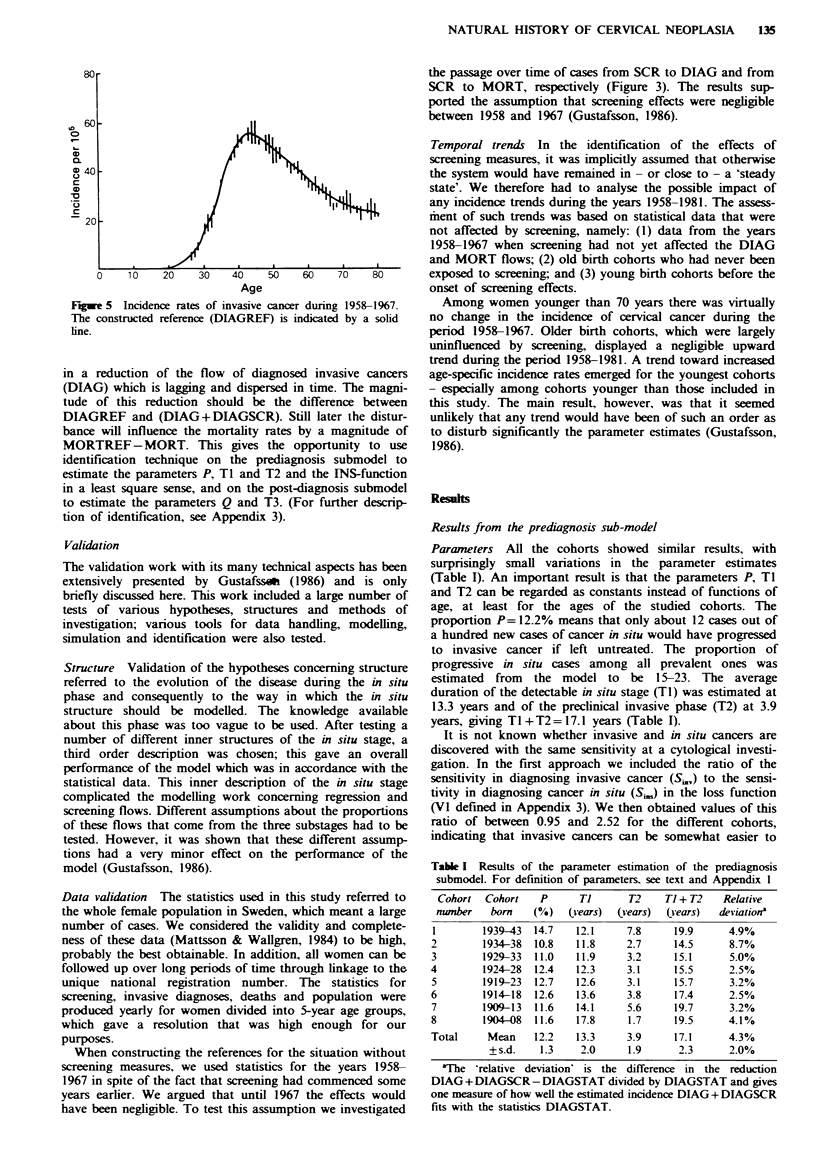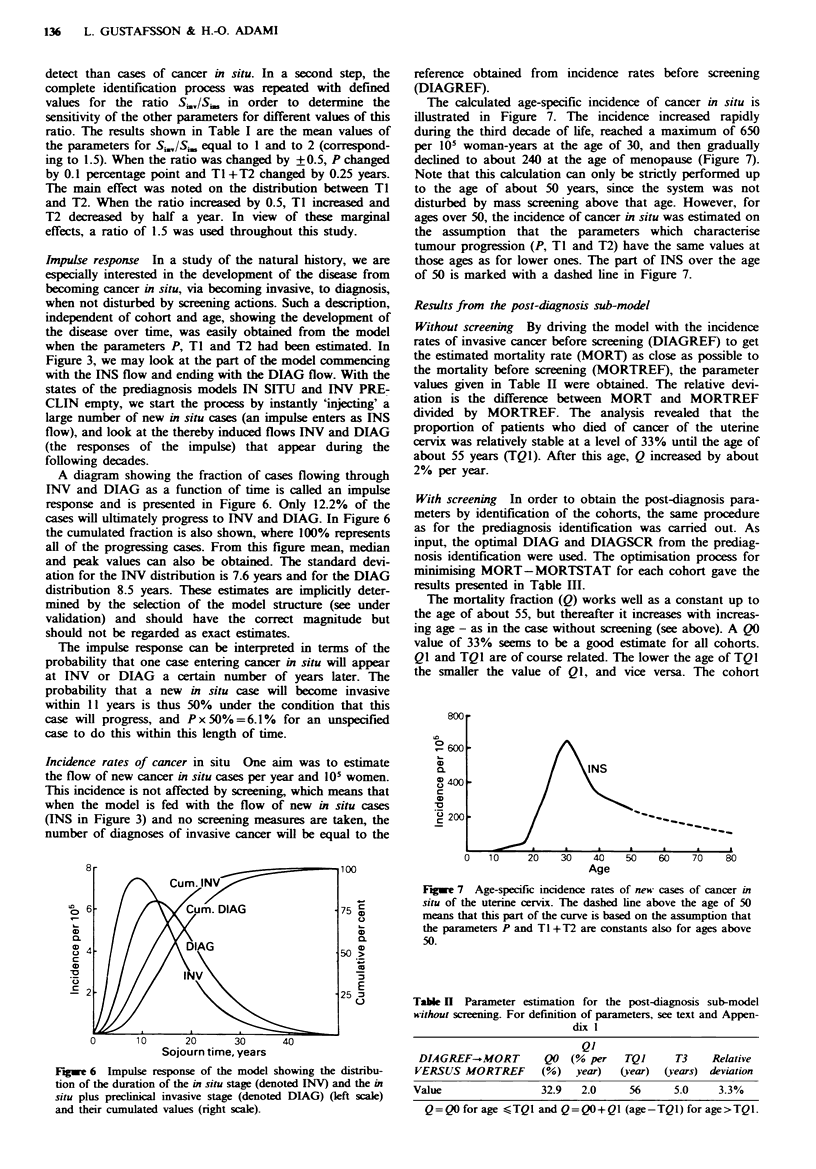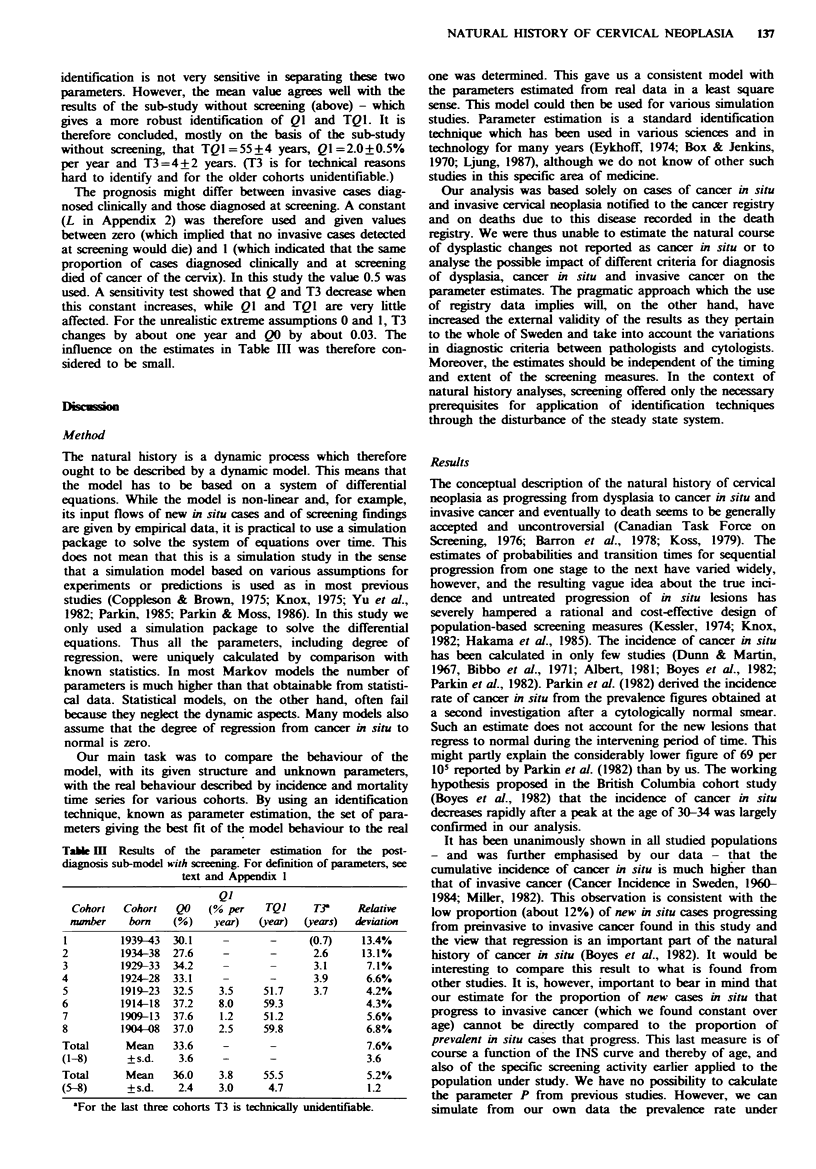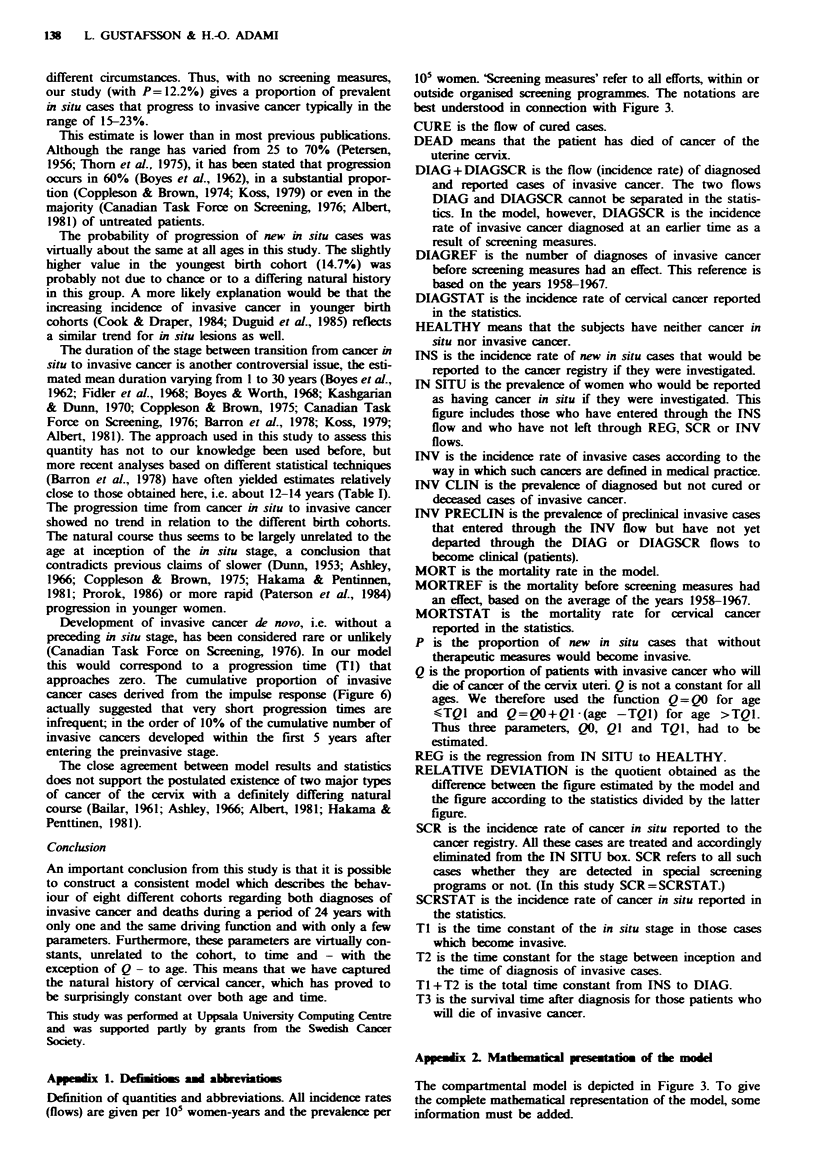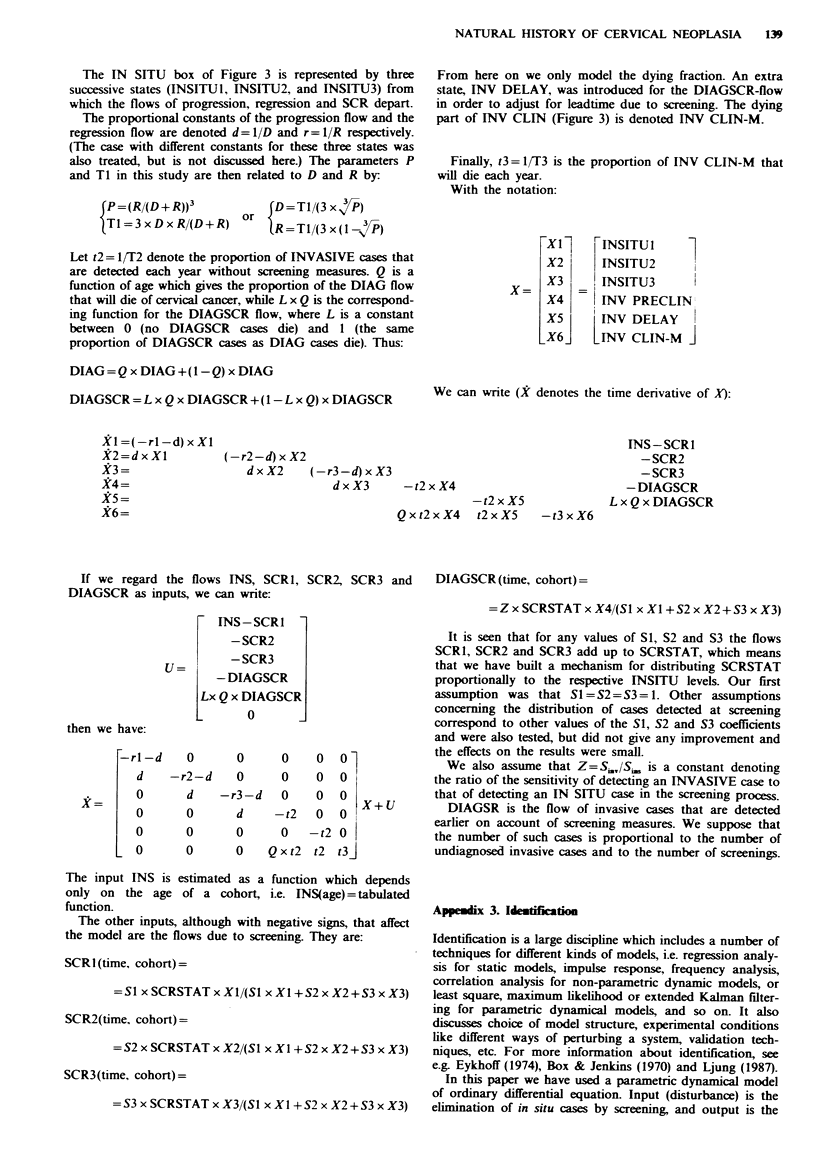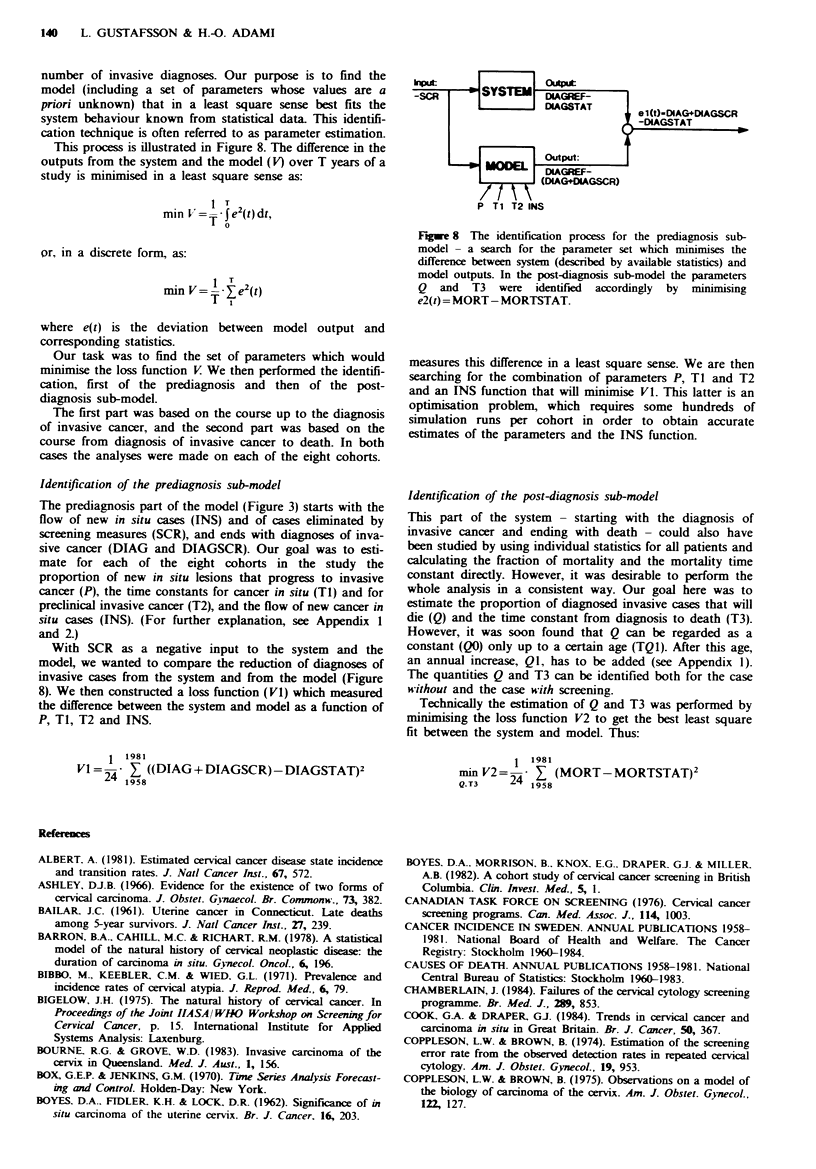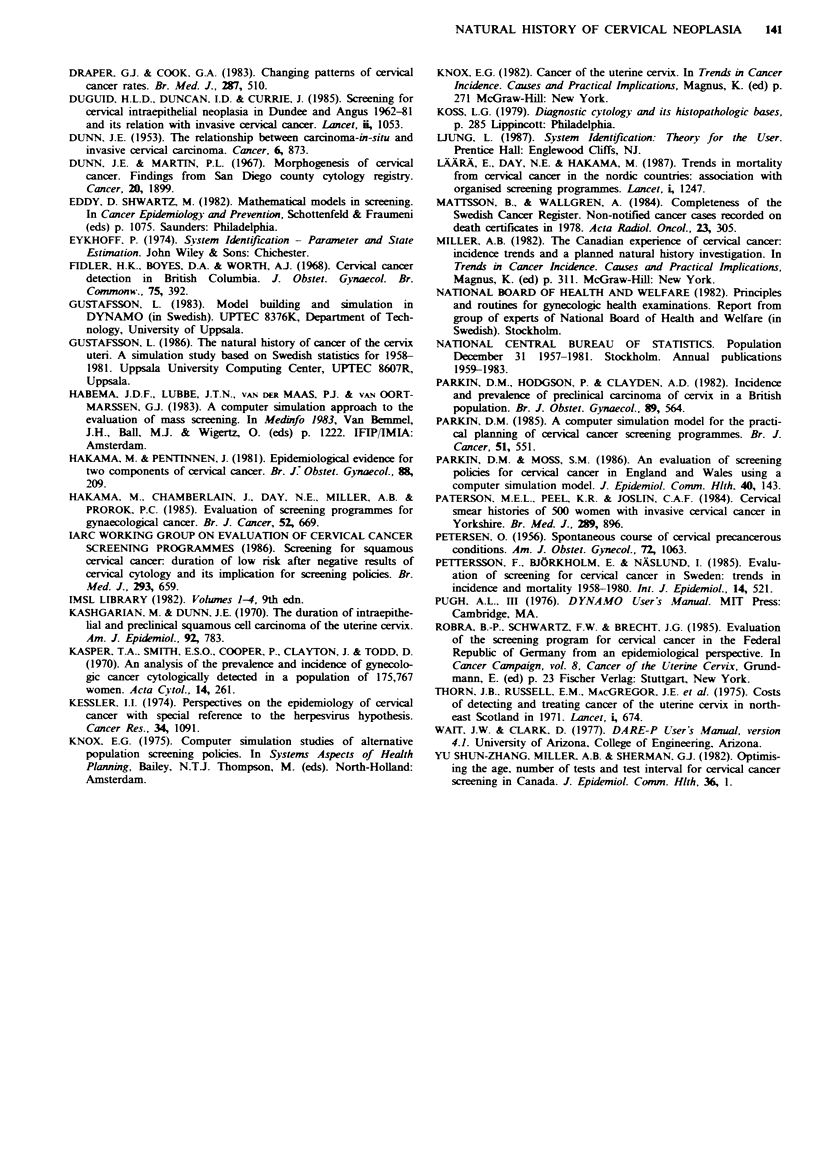# Natural history of cervical neoplasia: consistent results obtained by an identification technique.

**DOI:** 10.1038/bjc.1989.236

**Published:** 1989-07

**Authors:** L. Gustafsson, H. O. Adami

**Affiliations:** Teknikum, Uppsala University, Sweden.

## Abstract

Swedish population-based incidence and mortality rates for cancer of the uterine cervix, both in situ and invasive, during the period 1958 to 1981 were determined by means of a dynamic model. This new approach describes without any preconceptions the development of the disease as a sequential process over the stages cancer in situ, invasive cancer before and after diagnosis, and death. The strong disturbance of the steady-state situation that occurred after the introduction of cytological mass screening in the early 1960s permitted the use of a computerized identification technique. The whole natural history of cervical cancer could thus be identified and described consistently, with the mutual compatibility between statistical data, structure, parameters, and the states and flows between the states. The estimated age-specific incidence of cancer in situ increased rapidly to a maximum of 650 per 10(5) woman-years at the age of 30 years, after which it declined, and that of invasive cancer to a maximum of 55 per 10(5) at the age of 43. The natural history of cervical neoplasia did not differ appreciably between eight successive 5-year birth cohorts. The proportion of cases of new cancer in situ that progressed to invasive cancer was 12.2%, with a mean duration of the in situ stage in these cases of 13.3 years. The preclinical phase of the invasive stage (without screening) lasted on average about 4 years.


Br. J. Cancer (1989), 60, 132-141 © The Macmillan Press Ltd., 1989

### Natural history of cervical neoplasia: consistent results obtained by an identification technique

L. Gustafsson[1] & H.-O. Adami[2]

[1]*Teknikum, Uppsala University, Box 534, S-751 21 Uppsala, Sweden; and* [2]*Department of Surgery, University Hospital, S-751 85 Uppsala, Sweden.*

**Summary** Swedish population-based incidence and mortality rates for cancer of the uterine cervix, both *in situ* and invasive, during the period 1958 to 1981 were determined by means of a dynamic model. This new approach describes without any preconceptions the development of the disease as a sequential process over the stages cancer *in situ*, invasive cancer before and after diagnosis, and death. The strong disturbance of the steady-state situation that occurred after the introduction of cytological mass screening in the early 1960s permitted the use of a computerized identification technique. The whole natural history of cervical cancer could thus be identified and described consistently, with the mutual compatibility between statistical data, structure, parameters, and the states and flows between the states. The estimated age-specific incidence of cancer *in situ* increased rapidly to a maximum of 650 per $10^5$ woman-years at the age of 30 years, after which it declined, and that of invasive cancer to a maximum of 55 per $10^5$ at the age of 43. The natural history of cervical neoplasia did not differ appreciably between eight successive 5-year birth cohorts. The proportion of cases of new cancer *in situ* that progressed to invasive cancer was 12.2%, with a mean duration of the *in situ* stage in these cases of 13.3 years. The preclinical phase of the invasive stage (without screening) lasted on average about 4 years.

Cytological screening for cancer of the uterine cervix has been widely performed throughout the western world since the early 1960s (Canadian Task Force on Screening, 1976; Bourne & Grove, 1983; Hakama *et al.*, 1985; Duguid *et al.*, 1985; Pettersson *et al.*, 1985; Robra *et al.*, 1985; Läärä *et al.*, 1987) and it is now generally accepted that the mortality rate from cancer of the cervix has declined as a result of the screening measures (Kessler, 1974; Bourne & Grove, 1983; Duguid *et al.*, 1985; Hakama *et al.*, 1985; Pettersson *et al.*, 1985; Robra *et al.*, 1985; IARC Working Group, 1986). This conclusion, however, remained controversial during many years for two major reasons, namely the lack of randomised trials and the deficient knowledge about the natural history of cervical neoplasia. It seems to be generally accepted that invasive cancer of the uterine cervix is preceded in most or all instances by a preinvasive (*in situ*) stage (Boyes *et al.*, 1982). Data on the age-specific incidence of cancer *in situ* are largely lacking and there are only few and contradictory estimates of the proportion of *in situ* cancers that actually progress to the invasive form and of the time constants in the evolution from preinvasive cancer to clinical diagnosis (Kessler, 1974; Boyes *et al.*, 1982; Knox, 1982). It is also unknown whether these parameters are associated with the woman's age.

Beyond its obvious interest from a tumour-biological point of view, a clear picture of the natural history of cervical neoplasia would probably facilitate cost-effective designing of screening interventions and might eventually minimise overtreatment of preinvasive cancers that otherwise would not have progressed to an invasive stage. In most instances, however, ethical constraints prohibit studies on the untreated progress of premalignant and malignant diseases. Advancement of knowledge in this area – which has even been considered impossible (Kessler, 1974; Draper & Cook, 1983) – therefore requires other approaches than those traditionally used in medical research (Knox, 1982). Examples of such alternative approaches are simulation techniques (Bigelow, 1975; Coppleson & Brown, 1975; Knox, 1975; Barron *et al.*, 1978; Eddy & Shwartz, 1982; Yu *et al.*, 1982; Habema *et al.*, 1983, Parkin, 1985; Parkin & Moss, 1986).

From the field of control theory it is well known that the parameters of a system can be calculated provided that its structure is known and that the system is disturbed. In this study, the access to suitable simulation languages and mathematical packages (Pugh, 1976; Wait & Clark, 1977; The IMSL Library, 1982; Gustafsson, 1983) facilitated our approach which consisted of the use of an identification technique. In addition, favourable prerequisites were offered in Sweden through the availability of reliable incidence (Cancer Incidence in Sweden, 1960–1984) and mortality (Causes of Death 1958–1981) rates for cancer of the uterine cervix over a 24-year period for the entire population of about four million women, and through the extensive screening programme that was introduced in Sweden during the early 1960s (National Board of Health and Welfare, 1982).

The purpose of this study was to investigate the natural history of cervical neoplasia in terms of the states cancer *in situ*, preclinical invasive cancer and invasive cancer after diagnosis, and death, and the flows to, between and from these states. A dynamic model which describes how these states and flows are related was constructed.

We have tried to facilitate the reading of this paper by keeping the presentation of technical aspects at a minimum and presenting them in appendices. Detailed information may, however, be of decisive importance for a critical assessment of the internal validity of the study. An extensive methodological presentation is therefore available on request (Gustafsson, 1986).

### Background and material

### *Cytological screening*

Cytological screening for cervical cancer with Papanicolaou smears started in Sweden in the early 1960s, and organised population-based programmes were successively introduced during the period 1967–1973 (National Board of Health and Welfare, 1982; Pettersson *et al.*, 1985). Women aged 30–49 years were invited to undergo cytological screening at 4-year intervals. The extent of the organised screening was limited, however, and the rate of participation was low until the end of the 1960s, with only about 9,000 examinations in 1967. The total annual number of smears – within and outside the organised screening – then increased rapidly to about one million in 1970 and thereafter (National Board of Health and Welfare, 1982). Less than one-quarter of the smears have been taken within the organised screening and the remainder outside this scheme at hospitals and outpatient

Correspondence: L. Gustafsson.

clinics (National Board of Health and Welfare, 1982). By all these means, which are denoted 'screening measures' in the following text, a large number of cases of cancer *in situ* have been detected (Figure 1) and cured every year. This intervention therefore constitutes a disturbance of a steady state situation, resulting in a reduction in the number of cases of diagnosed invasive cancer some years later, and eventually a reduction in the number of deaths due to cervical cancer.

**Figure 1** The total annual number of cases of cancer *in situ* and invasive cancers of the uterine cervix reported to the Swedish Cancer Registry and of deaths due to cervical cancer reported to the Death Registry during the period 1958–1981.

**Figure 2** The age-specific incidence rates of invasive cancer of the uterine cervix during defined years.

*The cancer registry*

Sweden has a population-based cancer registry which has been operating since 1958 (Cancer Incidence in Sweden, 1960–1984). It is obligatory for all physicians in hospitals and other establishments for medical treatment in Sweden to report to the National Cancer Registry all cases of diagnosed cancer. In addition, pathologists and cytologists separately report every cancer diagnosis based on surgically removed tissues, biopsies, cytological specimens and autopsies to the registry. This obligation to report also includes cancer *in situ* of the cervix uteri. The frequency of underreporting to the registry has been estimated to be 0.7% for cancers of the female genital organs (Mattsson & Wallgren, 1984). Swedish population-based mortality statistics, which are published annually, cover the entire period of cancer registration (Causes of Death, 1960–1983). The cancer registry is linked annually to the death registry and the date and causes of death are transferred.

Four types of statistical data, based on the entire Swedish female population, were used and divided into 5-year age groups and are presented for each year. Thus we have used annual figures for the years 1958–1981 for calculating: (1) the number of detected new cases of cancer *in situ* of the cervix uteri (Cancer Incidence in Sweden, 1960–1984); (2) the number of detected new cases of invasive cancer of the cervix. (Cancer Incidence in Sweden, 1960–1984); (3) the number of deaths due to cervical cancer (Causes of Death, 1960–1983); (4) the number of women of different ages (National Central Bureau of Statistics, 1959–1983).

During the period 1958–1981 the total number of reported cases of cancer *in situ* was 64,215, of invasive cancer 18,218 and of deaths due to cancer of the uterine cervix 6,990 (Figure 1). From the population statistics the number of women at risk was calculated as the average of the number at the beginning and at the end of each year.

The number of detected cases with cancer *in situ* and with invasive cancer and the number of deaths per year are shown in Figure 1. After the end of the 1960s the incidence of *in situ* cases found at screening was about five times that of invasive cases diagnosed before screening measures were undertaken, showing that only a small proportion of the cancer *in situ* cases progressed. The surplus number of invasive cancer and the number of deaths per year are shown detection at screening is also evident (Figure 1). The screening effects are illustrated by age in Figure 2, which shows a successive reduction from 1965 to 1981 in the incidence of invasive cancer between the ages of 30 and 65 years.

### Methods

To analyse the dynamics from the healthy state via cancer *in situ* and invasive cancer to death, we need (1) a structural

**Figure 3** The natural history of cervical neoplasia as a compartmental model. Transitions between states (□) take place by flows (→), which are controlled by rate valves (⧖). All the flows have the dimension of number of cases per $10^5$ women-years. The flows for which statistics are available from cancer and death registers are indicated by filled valves (⧗). Abbreviations and parameters are explained in the text and in Appendix 1.

description (model) of the natural history based on differential equations, (2) appropriate statistics describing this form of cancer during a time period longer than the time constants of the system and (3) an identification method to calculate the unknown quantities of the model from the statistical data.

*Model structure*

Cervical cancer evolves with time from the state of being healthy or having dysplasia, via cancer *in situ* and invasive cancer, to death. We have some branching flows from this sequence of progression of the disease. Thus *in situ* cases may progress, remain stationary, regress or be discovered at screening and cured. Diagnosed invasive cases may also be cured. The main conceptual evolution is now elaborated into a dynamic, integrated, compartmental model (Figure 3 and Appendix 2). The states (prevalences) are represented by levels or numbers of cases per $10^5$ women. They change only as a result of flows (incidences), which are numbers of cases per year and $10^5$ women. Both states and flows vary with time. The designations in Figure 3 are defined in Appendix 1 and will be used in the following text in order to be exact and avoid confusion.

The number of *in situ* cases that are detected (SCR) is highly dependent on the extent of cytological screening, and the true incidence of cancer *in situ* (INS) is unknown. The *in situ* box has a more detailed inner structure than is shown in Figure 3. After extensive studies, a three-box structure was found to give the most appropriate description of the *in situ* stage. These investigations are discussed further below.

Invasive cancers have a preclinical phase during which nothing is known and registered and – after detection – a clinical phase during which the woman is a patient and can be followed up (Figure 3). In the absence of screening, most diagnosed cervical neoplasms are in the invasive stage, whereas during screening a much greater number of *in situ* (SCR) than of invasive (DIAG+DIAGSCR) cancers are detected. The incidence flows of invasive cancers that surface clinically (DIAG) and those of such cancers that are detected at screening (DIAGSCR) cannot be separated on the basis of the statistics from the Cancer Registry (Cancer Incidence in Sweden, 1960–1984). It is these invasive cancers which were found earlier as a result of screening that cause the 'bump' in the invasive curve of Figure 1.

The model also includes a number of unknown parameters. These are the sojourn times: T1, T2 and T3 in the states of IN SITU, INV PRECLIN and INV CLIN respectively, and the proportion ($P$) of *new in situ* lesions that without therapeutic measures would become invasive, and finally the proportion ($Q$) of patients with invasive cancer who will die of cancer of the cervix uteri. These parameters are also shown in Figure 3 and exactly defined in Appendices 1 and 2.

Estimating these parameters gives a unique model by which all the flows and states can easily be calculated.

*Statistical preprocessing*

*Cohort data* The original data, obtained from official Swedish statistics (Causes of Death, 1960–1983; National Central Bureau of Statistics, 1959–1983; Cancer Incidence in Sweden, 1960–1984) for each year and each 5-year age group were compiled and entered into matrices of *in situ* and invasive diagnoses, death and population. For technical reasons it was appropriate to divide the *in situ* and invasive diagnoses and death matrices by the population matrix and multiply by $10^5$, which meant that we got three age-specific matrices. In this context we wanted to distort the information as little as possible and obtain smoothed data that were unbiased over time. For this reason a 3-year average was used, creating a new matrix from the old one, where each new value was obtained as:

$$X_{new}(t) = (X(t-1) + X(t) + X(t+1))/3.$$

This means that the three terms $X(t-1)$, $X(t)$, and $X(t+1)$ all refer to the same age but are sampled in three consecutive years.

In order to study the effects of screening, which commenced in Sweden in the mid-sixties, we chose to analyse the eight 5-year birth cohorts born between 1904 and 1943. Of these cohorts, the six youngest had been heavily exposed to screening measures, whereas the two oldest had been only slightly exposed. The eight cohorts were of ages 50–54, 45–49 and so on in 1958, with the youngest 15–19 years old. They represented about two million women, including totals of 38,969 women with cancer *in situ*, 13,830 women with invasive cancer and 4,784 deaths due to cancer of the uterine cervix during the 24-year study period. As a result we now had the necessary statistics, which we denote SCRSTAT for diagnoses of cancer *in situ*, DIAGSTAT for diagnoses of invasive cancer and MORTSTAT for mortality from cervical cancer, expressed per $10^5$ women-years as based on cohort data. In Figure 4 the numbers of diagnoses of invasive cancer from 1958 through 1981 are shown for the eight cohorts of this study.

*Steady-state references* To calculate the way in which screening affects the annual number of diagnosed cases of invasive cancer and of deaths, information was required both for the situation *without* and for that *with* screening. We therefore first calculated, as references, the annual numbers for the situation when the system was not disturbed by screening measures.

*Reference for incidence rates* It is evident from the incidence rates of cancer *in situ* in the Cancer Registry that between 1958 and 1963 the degree of screening was insignificant (Figure 1). Moreover, only few of those cases detected at screening during 1964–1967 would otherwise have been diagnosed as invasive cancer before 1968. Screening thus affected the incidence rates of invasive cancer to only a negligible extent between 1958 and 1967. The incidence rates for invasive cancer (Figure 4) were then plotted against age for each cohort during only the first 10-year period 1958–1967 of no screening effects. This resulted in a diagram where each year is represented by values from exactly two of the 5-year cohorts. Connecting these two values of each year gives the vertical bars of Figure 5. In this figure the reference (DIAGREF) of age-specific incidence rates was constructed and denoted with a solid line.

*Reference for mortality rates* In the same way as DIAGREF (see above), a reference for mortality rates (MORTREF) was derived from the mortality rates for the cohorts (MORTSTAT) in 1958–1967.

*Identification*

The way in which screening measures affect the whole system is illustrated in Figure 3. The reported cancer *in situ* cases (SCR) were subtracted from the *in situ* box, resulting

**Figure 4** Incidence of invasive cancer expressed as annual rates per $10^5$ women by age and year of birth (birth cohorts).

**Figure 5** Incidence rates of invasive cancer during 1958–1967. The constructed reference (DIAGREF) is indicated by a solid line.

in a reduction of the flow of diagnosed invasive cancers (DIAG) which is lagging and dispersed in time. The magnitude of this reduction should be the difference between DIAGREF and (DIAG + DIAGSCR). Still later the disturbance will influence the mortality rates by a magnitude of MORTREF − MORT. This gives the opportunity to use identification technique on the prediagnosis submodel to estimate the parameters $P$, T1 and T2 and the INS-function in a least square sense, and on the post-diagnosis submodel to estimate the parameters $Q$ and T3. (For further description of identification, see Appendix 3).

*Validation*

The validation work with its many technical aspects has been extensively presented by Gustafsson (1986) and is only briefly discussed here. This work included a large number of tests of various hypotheses, structures and methods of investigation; various tools for data handling, modelling, simulation and identification were also tested.

*Structure* Validation of the hypotheses concerning structure referred to the evolution of the disease during the *in situ* phase and consequently to the way in which the *in situ* structure should be modelled. The knowledge available about this phase was too vague to be used. After testing a number of different inner structures of the *in situ* stage, a third order description was chosen; this gave an overall performance of the model which was in accordance with the statistical data. This inner description of the *in situ* stage complicated the modelling work concerning regression and screening flows. Different assumptions about the proportions of these flows that come from the three substages had to be tested. However, it was shown that these different assumptions had a very minor effect on the performance of the model (Gustafsson, 1986).

*Data validation* The statistics used in this study referred to the whole female population in Sweden, which meant a large number of cases. We considered the validity and completeness of these data (Mattsson & Wallgren, 1984) to be high, probably the best obtainable. In addition, all women can be followed up over long periods of time through linkage to the unique national registration number. The statistics for screening, invasive diagnoses, deaths and population were produced yearly for women divided into 5-year age groups, which gave a resolution that was high enough for our purposes.

When constructing the references for the situation without screening measures, we used statistics for the years 1958–1967 in spite of the fact that screening had commenced some years earlier. We argued that until 1967 the effects would have been negligible. To test this assumption we investigated the passage over time of cases from SCR to DIAG and from SCR to MORT, respectively (Figure 3). The results supported the assumption that screening effects were negligible between 1958 and 1967 (Gustafsson, 1986).

*Temporal trends* In the identification of the effects of screening measures, it was implicitly assumed that otherwise the system would have remained in – or close to – a 'steady state'. We therefore had to analyse the possible impact of any incidence trends during the years 1958–1981. The assessment of such trends was based on statistical data that were not affected by screening, namely: (1) data from the years 1958–1967 when screening had not yet affected the DIAG and MORT flows; (2) old birth cohorts who had never been exposed to screening; and (3) young birth cohorts before the onset of screening effects.

Among women younger than 70 years there was virtually no change in the incidence of cervical cancer during the period 1958–1967. Older birth cohorts, which were largely uninfluenced by screening, displayed a negligible upward trend during the period 1958–1981. A trend toward increased age-specific incidence rates emerged for the youngest cohorts – especially among cohorts younger than those included in this study. The main result, however, was that it seemed unlikely that any trend would have been of such an order as to disturb significantly the parameter estimates (Gustafsson, 1986).

### Results

*Results from the prediagnosis sub-model*

*Parameters* All the cohorts showed similar results, with surprisingly small variations in the parameter estimates (Table I). An important result is that the parameters $P$, T1 and T2 can be regarded as constants instead of functions of age, at least for the ages of the studied cohorts. The proportion $P = 12.2\%$ means that only about 12 cases out of a hundred new cases of cancer *in situ* would have progressed to invasive cancer if left untreated. The proportion of progressive *in situ* cases among all prevalent ones was estimated from the model to be 15–23. The average duration of the detectable *in situ* stage (T1) was estimated at 13.3 years and of the preclinical invasive phase (T2) at 3.9 years, giving T1 + T2 = 17.1 years (Table I).

It is not known whether invasive and *in situ* cancers are discovered with the same sensitivity at a cytological investigation. In the first approach we included the ratio of the sensitivity in diagnosing invasive cancer ($S_{inv}$) to the sensitivity in diagnosing cancer *in situ* ($S_{ins}$) in the loss function (V1 defined in Appendix 3). We then obtained values of this ratio of between 0.95 and 2.52 for the different cohorts, indicating that invasive cancers can be somewhat easier to

**Table I** Results of the parameter estimation of the prediagnosis submodel. For definition of parameters, see text and Appendix 1

| *Cohort number* | *Cohort born* | *P* (%) | *T1* (*years*) | *T2* (*years*) | *T1 + T2* (*years*) | *Relative deviation*[a] |
|---|---|---|---|---|---|---|
| 1 | 1939–43 | 14.7 | 12.1 | 7.8 | 19.9 | 4.9% |
| 2 | 1934–38 | 10.8 | 11.8 | 2.7 | 14.5 | 8.7% |
| 3 | 1929–33 | 11.0 | 11.9 | 3.2 | 15.1 | 5.0% |
| 4 | 1924–28 | 12.4 | 12.3 | 3.1 | 15.5 | 2.5% |
| 5 | 1919–23 | 12.7 | 12.6 | 3.1 | 15.7 | 3.2% |
| 6 | 1914–18 | 12.6 | 13.6 | 3.8 | 17.4 | 2.5% |
| 7 | 1909–13 | 11.6 | 14.1 | 5.6 | 19.7 | 3.2% |
| 8 | 1904–08 | 11.6 | 17.8 | 1.7 | 19.5 | 4.1% |
| Total | Mean | 12.2 | 13.3 | 3.9 | 17.1 | 4.3% |
| | ±s.d. | 1.3 | 2.0 | 1.9 | 2.3 | 2.0% |

[a]The 'relative deviation' is the difference in the reduction DIAG + DIAGSCR − DIAGSTAT divided by DIAGSTAT and gives one measure of how well the estimated incidence DIAG + DIAGSCR fits with the statistics DIAGSTAT.

detect than cases of cancer *in situ*. In a second step, the complete identification process was repeated with defined values for the ratio $S_{inv}/S_{ins}$ in order to determine the sensitivity of the other parameters for different values of this ratio. The results shown in Table I are the mean values of the parameters for $S_{inv}/S_{ins}$ equal to 1 and to 2 (corresponding to 1.5). When the ratio was changed by $\pm 0.5$, $P$ changed by 0.1 percentage point and T1 + T2 changed by 0.25 years. The main effect was noted on the distribution between T1 and T2. When the ratio increased by 0.5, T1 increased and T2 decreased by half a year. In view of these marginal effects, a ratio of 1.5 was used throughout this study.

*Impulse response* In a study of the natural history, we are especially interested in the development of the disease from becoming cancer *in situ*, via becoming invasive, to diagnosis, when not disturbed by screening actions. Such a description, independent of cohort and age, showing the development of the disease over time, was easily obtained from the model when the parameters $P$, T1 and T2 had been estimated. In Figure 3, we may look at the part of the model commencing with the INS flow and ending with the DIAG flow. With the states of the prediagnosis models IN SITU and INV PRECLIN empty, we start the process by instantly 'injecting' a large number of new *in situ* cases (an impulse enters as INS flow), and look at the thereby induced flows INV and DIAG (the responses of the impulse) that appear during the following decades.

A diagram showing the fraction of cases flowing through INV and DIAG as a function of time is called an impulse response and is presented in Figure 6. Only 12.2% of the cases will ultimately progress to INV and DIAG. In Figure 6 the cumulated fraction is also shown, where 100% represents all of the progressing cases. From this figure mean, median and peak values can also be obtained. The standard deviation for the INV distribution is 7.6 years and for the DIAG distribution 8.5 years. These estimates are implicitly determined by the selection of the model structure (see under validation) and should have the correct magnitude but should not be regarded as exact estimates.

The impulse response can be interpreted in terms of the probability that one case entering cancer *in situ* will appear at INV or DIAG a certain number of years later. The probability that a new *in situ* case will become invasive within 11 years is thus 50% under the condition that this case will progress, and $P \times 50\% = 6.1\%$ for an unspecified case to do this within this length of time.

*Incidence rates of cancer* in situ One aim was to estimate the flow of new cancer *in situ* cases per year and $10^5$ women. This incidence is not affected by screening, which means that when the model is fed with the flow of new *in situ* cases (INS in Figure 3) and no screening measures are taken, the number of diagnoses of invasive cancer will be equal to the reference obtained from incidence rates before screening (DIAGREF).

The calculated age-specific incidence of cancer *in situ* is illustrated in Figure 7. The incidence increased rapidly during the third decade of life, reached a maximum of 650 per $10^5$ woman-years at the age of 30, and then gradually declined to about 240 at the age of menopause (Figure 7). Note that this calculation can only be strictly performed up to the age of about 50 years, since the system was not disturbed by mass screening above that age. However, for ages over 50, the incidence of cancer *in situ* was estimated on the assumption that the parameters which characterise tumour progression ($P$, T1 and T2) have the same values at those ages as for lower ones. The part of INS over the age of 50 is marked with a dashed line in Figure 7.

*Results from the post-diagnosis sub-model*

*Without screening* By driving the model with the incidence rates of invasive cancer before screening (DIAGREF) to get the estimated mortality rate (MORT) as close as possible to the mortality before screening (MORTREF), the parameter values given in Table II were obtained. The relative deviation is the difference between MORT and MORTREF divided by MORTREF. The analysis revealed that the proportion of patients who died of cancer of the uterine cervix was relatively stable at a level of 33% until the age of about 55 years ($TQ1$). After this age, $Q$ increased by about 2% per year.

*With screening* In order to obtain the post-diagnosis parameters by identification of the cohorts, the same procedure as for the prediagnosis identification was carried out. As input, the optimal DIAG and DIAGSCR from the prediagnosis identification were used. The optimisation process for minimising MORT − MORTSTAT for each cohort gave the results presented in Table III.

The mortality fraction ($Q$) works well as a constant up to the age of about 55, but thereafter it increases with increasing age – as in the case without screening (see above). A $Q0$ value of 33% seems to be a good estimate for all cohorts. $Q1$ and $TQ1$ are of course related. The lower the age of $TQ1$ the smaller the value of $Q1$, and vice versa. The cohort

**Figure 7** Age-specific incidence rates of *new* cases of cancer *in situ* of the uterine cervix. The dashed line above the age of 50 means that this part of the curve is based on the assumption that the parameters $P$ and T1 + T2 are constants also for ages above 50.

**Figure 6** Impulse response of the model showing the distribution of the duration of the *in situ* stage (denoted INV) and the *in situ* plus preclinical invasive stage (denoted DIAG) (left scale) and their cumulated values (right scale).

**Table II** Parameter estimation for the post-diagnosis sub-model *without* screening. For definition of parameters, see text and Appendix 1

| *DIAGREF→MORT VERSUS MORTREF* | *Q0 (%)* | *Q1 (% per year)* | *TQ1 (year)* | *T3 (years)* | *Relative deviation* |
|---|---|---|---|---|---|
| Value | 32.9 | 2.0 | 56 | 5.0 | 3.3% |

$Q = Q0$ for age $\leqslant TQ1$ and $Q = Q0 + Q1$ (age − $TQ1$) for age $> TQ1$.

identification is not very sensitive in separating these two parameters. However, the mean value agrees well with the results of the sub-study without screening (above) – which gives a more robust identification of $Q1$ and $TQ1$. It is therefore concluded, mostly on the basis of the sub-study without screening, that $TQ1 = 55 \pm 4$ years, $Q1 = 2.0 \pm 0.5\%$ per year and $T3 = 4 \pm 2$ years. (T3 is for technical reasons hard to identify and for the older cohorts unidentifiable.)

The prognosis might differ between invasive cases diagnosed clinically and those diagnosed at screening. A constant ($L$ in Appendix 2) was therefore used and given values between zero (which implied that no invasive cases detected at screening would die) and 1 (which indicated that the same proportion of cases diagnosed clinically and at screening died of cancer of the cervix). In this study the value 0.5 was used. A sensitivity test showed that $Q$ and T3 decrease when this constant increases, while $Q1$ and $TQ1$ are very little affected. For the unrealistic extreme assumptions 0 and 1, T3 changes by about one year and $Q0$ by about 0.03. The influence on the estimates in Table III was therefore considered to be small.

### Discussion

### *Method*

The natural history is a dynamic process which therefore ought to be described by a dynamic model. This means that the model has to be based on a system of differential equations. While the model is non-linear and, for example, its input flows of new *in situ* cases and of screening findings are given by empirical data, it is practical to use a simulation package to solve the system of equations over time. This does not mean that this is a simulation study in the sense that a simulation model based on various assumptions for experiments or predictions is used as in most previous studies (Coppleson & Brown, 1975; Knox, 1975; Yu *et al.*, 1982; Parkin, 1985; Parkin & Moss, 1986). In this study we only used a simulation package to solve the differential equations. Thus all the parameters, including degree of regression, were uniquely calculated by comparison with known statistics. In most Markov models the number of parameters is much higher than that obtainable from statistical data. Statistical models, on the other hand, often fail because they neglect the dynamic aspects. Many models also assume that the degree of regression from cancer *in situ* to normal is zero.

Our main task was to compare the behaviour of the model, with its given structure and unknown parameters, with the real behaviour described by incidence and mortality time series for various cohorts. By using an identification technique, known as parameter estimation, the set of parameters giving the best fit of the model behaviour to the real one was determined. This gave us a consistent model with the parameters estimated from real data in a least square sense. This model could then be used for various simulation studies. Parameter estimation is a standard identification technique which has been used in various sciences and in technology for many years (Eykhoff, 1974; Box & Jenkins, 1970; Ljung, 1987), although we do not know of other such studies in this specific area of medicine.

Our analysis was based solely on cases of cancer *in situ* and invasive cervical neoplasia notified to the cancer registry and on deaths due to this disease recorded in the death registry. We were thus unable to estimate the natural course of dysplastic changes not reported as cancer *in situ* or to analyse the possible impact of different criteria for diagnosis of dysplasia, cancer *in situ* and invasive cancer on the parameter estimates. The pragmatic approach which the use of registry data implies will, on the other hand, have increased the external validity of the results as they pertain to the whole of Sweden and take into account the variations in diagnostic criteria between pathologists and cytologists. Moreover, the estimates should be independent of the timing and extent of the screening measures. In the context of natural history analyses, screening offered only the necessary prerequisites for application of identification techniques through the disturbance of the steady state system.

**Table III** Results of the parameter estimation for the post-diagnosis sub-model *with* screening. For definition of parameters, see text and Appendix 1

| *Cohort number* | *Cohort born* | *Q0 (%)* | *Q1 (% per year)* | *TQ1 (year)* | *T3*^a^ *(years)* | *Relative deviation* |
|---|---|---|---|---|---|---|
| 1 | 1939–43 | 30.1 | – | – | (0.7) | 13.4% |
| 2 | 1934–38 | 27.6 | – | – | 2.6 | 13.1% |
| 3 | 1929–33 | 34.2 | – | – | 3.1 | 7.1% |
| 4 | 1924–28 | 33.1 | – | – | 3.9 | 6.6% |
| 5 | 1919–23 | 32.5 | 3.5 | 51.7 | 3.7 | 4.2% |
| 6 | 1914–18 | 37.2 | 8.0 | 59.3 | | 4.3% |
| 7 | 1909–13 | 37.6 | 1.2 | 51.2 | | 5.6% |
| 8 | 1904–08 | 37.0 | 2.5 | 59.8 | | 6.8% |
| Total | Mean | 33.6 | – | – | | 7.6% |
| (1–8) | ±s.d. | 3.6 | – | – | | 3.6 |
| Total | Mean | 36.0 | 3.8 | 55.5 | | 5.2% |
| (5–8) | ±s.d. | 2.4 | 3.0 | 4.7 | | 1.2 |

[a]For the last three cohorts T3 is technically unidentifiable.

### *Results*

The conceptual description of the natural history of cervical neoplasia as progressing from dysplasia to cancer *in situ* and invasive cancer and eventually to death seems to be generally accepted and uncontroversial (Canadian Task Force on Screening, 1976; Barron *et al.*, 1978; Koss, 1979). The estimates of probabilities and transition times for sequential progression from one stage to the next have varied widely, however, and the resulting vague idea about the true incidence and untreated progression of *in situ* lesions has severely hampered a rational and cost-effective design of population-based screening measures (Kessler, 1974; Knox, 1982; Hakama *et al.*, 1985). The incidence of cancer *in situ* has been calculated in only few studies (Dunn & Martin, 1967, Bibbo *et al.*, 1971; Albert, 1981; Boyes *et al.*, 1982; Parkin *et al.*, 1982). Parkin *et al.* (1982) derived the incidence rate of cancer *in situ* from the prevalence figures obtained at a second investigation after a cytologically normal smear. Such an estimate does not account for the new lesions that regress to normal during the intervening period of time. This might partly explain the considerably lower figure of 69 per $10^5$ reported by Parkin *et al.* (1982) than by us. The working hypothesis proposed in the British Columbia cohort study (Boyes *et al.*, 1982) that the incidence of cancer *in situ* decreases rapidly after a peak at the age of 30–34 was largely confirmed in our analysis.

It has been unanimously shown in all studied populations – and was further emphasised by our data – that the cumulative incidence of cancer *in situ* is much higher than that of invasive cancer (Cancer Incidence in Sweden, 1960–1984; Miller, 1982). This observation is consistent with the low proportion (about 12%) of *new in situ* cases progressing from preinvasive to invasive cancer found in this study and the view that regression is an important part of the natural history of cancer *in situ* (Boyes *et al.*, 1982). It would be interesting to compare this result to what is found from other studies. It is, however, important to bear in mind that our estimate for the proportion of *new* cases *in situ* that progress to invasive cancer (which we found constant over age) cannot be directly compared to the proportion of *prevalent in situ* cases that progress. This last measure is of course a function of the INS curve and thereby of age, and also of the specific screening activity earlier applied to the population under study. We have no possibility to calculate the parameter $P$ from previous studies. However, we can simulate from our own data the prevalence rate under different circumstances. Thus, with no screening measures, our study (with $P=12.2\%$) gives a proportion of prevalent *in situ* cases that progress to invasive cancer typically in the range of 15–23%.

This estimate is lower than in most previous publications. Although the range has varied from 25 to 70% (Petersen, 1956; Thorn *et al.*, 1975), it has been stated that progression occurs in 60% (Boyes *et al.*, 1962), in a substantial proportion (Coppleson & Brown, 1974; Koss, 1979) or even in the majority (Canadian Task Force on Screening, 1976; Albert, 1981) of untreated patients.

The probability of progression of *new in situ* cases was virtually about the same at all ages in this study. The slightly higher value in the youngest birth cohort (14.7%) was probably not due to chance or to a differing natural history in this group. A more likely explanation would be that the increasing incidence of invasive cancer in younger birth cohorts (Cook & Draper, 1984; Duguid *et al.*, 1985) reflects a similar trend for *in situ* lesions as well.

The duration of the stage between transition from cancer *in situ* to invasive cancer is another controversial issue, the estimated mean duration varying from 1 to 30 years (Boyes *et al.*, 1962; Fidler *et al.*, 1968; Boyes & Worth, 1968; Kashgarian & Dunn, 1970; Coppleson & Brown, 1975; Canadian Task Force on Screening, 1976; Barron *et al.*, 1978; Koss, 1979; Albert, 1981). The approach used in this study to assess this quantity has not to our knowledge been used before, but more recent analyses based on different statistical techniques (Barron *et al.*, 1978) have often yielded estimates relatively close to those obtained here, i.e. about 12–14 years (Table I). The progression time from cancer *in situ* to invasive cancer showed no trend in relation to the different birth cohorts. The natural course thus seems to be largely unrelated to the age at inception of the *in situ* stage, a conclusion that contradicts previous claims of slower (Dunn, 1953; Ashley, 1966; Coppleson & Brown, 1975; Hakama & Pentinnen, 1981; Prorok, 1986) or more rapid (Paterson *et al.*, 1984) progression in younger women.

Development of invasive cancer *de novo*, i.e. without a preceding *in situ* stage, has been considered rare or unlikely (Canadian Task Force on Screening, 1976). In our model this would correspond to a progression time (T1) that approaches zero. The cumulative proportion of invasive cancer cases derived from the impulse response (Figure 6) actually suggested that very short progression times are infrequent; in the order of 10% of the cumulative number of invasive cancers developed within the first 5 years after entering the preinvasive stage.

The close agreement between model results and statistics does not support the postulated existence of two major types of cancer of the cervix with a definitely differing natural course (Bailar, 1961; Ashley, 1966; Albert, 1981; Hakama & Penttinen, 1981).

*Conclusion*

An important conclusion from this study is that it is possible to construct a consistent model which describes the behaviour of eight different cohorts regarding both diagnoses of invasive cancer and deaths during a period of 24 years with only one and the same driving function and with only a few parameters. Furthermore, these parameters are virtually constants, unrelated to the cohort, to time and – with the exception of $Q$ – to age. This means that we have captured the natural history of cervical cancer, which has proved to be surprisingly constant over both age and time.

This study was performed at Uppsala University Computing Centre and was supported partly by grants from the Swedish Cancer Society.

**Appendix 1. Definitions and abbreviations**

Definition of quantities and abbreviations. All incidence rates (flows) are given per $10^5$ women-years and the prevalence per $10^5$ women. 'Screening measures' refer to all efforts, within or outside organised screening programmes. The notations are best understood in connection with Figure 3.

CURE is the flow of cured cases.

DEAD means that the patient has died of cancer of the uterine cervix.

DIAG + DIAGSCR is the flow (incidence rate) of diagnosed and reported cases of invasive cancer. The two flows DIAG and DIAGSCR cannot be separated in the statistics. In the model, however, DIAGSCR is the incidence rate of invasive cancer diagnosed at an earlier time as a result of screening measures.

DIAGREF is the number of diagnoses of invasive cancer before screening measures had an effect. This reference is based on the years 1958–1967.

DIAGSTAT is the incidence rate of cervical cancer reported in the statistics.

HEALTHY means that the subjects have neither cancer *in situ* nor invasive cancer.

INS is the incidence rate of *new in situ* cases that would be reported to the cancer registry if they were investigated.

IN SITU is the prevalence of women who would be reported as having cancer *in situ* if they were investigated. This figure includes those who have entered through the INS flow and who have not left through REG, SCR or INV flows.

INV is the incidence rate of invasive cases according to the way in which such cancers are defined in medical practice.

INV CLIN is the prevalence of diagnosed but not cured or deceased cases of invasive cancer.

INV PRECLIN is the prevalence of preclinical invasive cases that entered through the INV flow but have not yet departed through the DIAG or DIAGSCR flows to become clinical (patients).

MORT is the mortality rate in the model.

MORTREF is the mortality before screening measures had an effect, based on the average of the years 1958–1967.

MORTSTAT is the mortality rate for cervical cancer reported in the statistics.

$P$ is the proportion of *new in situ* cases that without therapeutic measures would become invasive.

$Q$ is the proportion of patients with invasive cancer who will die of cancer of the cervix uteri. $Q$ is not a constant for all ages. We therefore used the function $Q=Q0$ for age $\leq TQ1$ and $Q=Q0+Q1\cdot(\text{age} - TQ1)$ for age $>TQ1$. Thus three parameters, $Q0$, $Q1$ and $TQ1$, had to be estimated.

REG is the regression from IN SITU to HEALTHY.

RELATIVE DEVIATION is the quotient obtained as the difference between the figure estimated by the model and the figure according to the statistics divided by the latter figure.

SCR is the incidence rate of cancer *in situ* reported to the cancer registry. All these cases are treated and accordingly eliminated from the IN SITU box. SCR refers to all such cases whether they are detected in special screening programs or not. (In this study SCR = SCRSTAT.)

SCRSTAT is the incidence rate of cancer *in situ* reported in the statistics.

T1 is the time constant of the *in situ* stage in those cases which become invasive.

T2 is the time constant for the stage between inception and the time of diagnosis of invasive cases.

T1 + T2 is the total time constant from INS to DIAG.

T3 is the survival time after diagnosis for those patients who will die of invasive cancer.

**Appendix 2. Mathematical presentation of the model**

The compartmental model is depicted in Figure 3. To give the complete mathematical representation of the model, some information must be added.

The IN SITU box of Figure 3 is represented by three successive states (INSITU1, INSITU2, and INSITU3) from which the flows of progression, regression and SCR depart.

The proportional constants of the progression flow and the regression flow are denoted $d=1/D$ and $r=1/R$ respectively. (The case with different constants for these three states was also treated, but is not discussed here.) The parameters $P$ and T1 in this study are then related to $D$ and $R$ by:

$$\begin{cases} P=(R/(D+R))^3 \\ \mathrm{T1}=3\times D\times R/(D+R) \end{cases} \quad \text{or} \quad \begin{cases} D=\mathrm{T1}/(3\times\sqrt[3]{P}) \\ R=\mathrm{T1}/(3\times(1-\sqrt[3]{P}) \end{cases}$$

Let $t2=1/\mathrm{T2}$ denote the proportion of INVASIVE cases that are detected each year without screening measures. $Q$ is a function of age which gives the proportion of the DIAG flow that will die of cervical cancer, while $L\times Q$ is the corresponding function for the DIAGSCR flow, where $L$ is a constant between 0 (no DIAGSCR cases die) and 1 (the same proportion of DIAGSCR cases as DIAG cases die). Thus:

$$\mathrm{DIAG}=Q\times\mathrm{DIAG}+(1-Q)\times\mathrm{DIAG}$$

$$\mathrm{DIAGSCR}=L\times Q\times\mathrm{DIAGSCR}+(1-L\times Q)\times\mathrm{DIAGSCR}$$

From here on we only model the dying fraction. An extra state, INV DELAY, was introduced for the DIAGSCR-flow in order to adjust for leadtime due to screening. The dying part of INV CLIN (Figure 3) is denoted INV CLIN-M.

Finally, $t3=1/\mathrm{T3}$ is the proportion of INV CLIN-M that will die each year.

With the notation:

$$X=\begin{bmatrix} X1 \\ X2 \\ X3 \\ X4 \\ X5 \\ X6 \end{bmatrix}=\begin{bmatrix} \mathrm{INSITU1} \\ \mathrm{INSITU2} \\ \mathrm{INSITU3} \\ \mathrm{INV\ PRECLIN} \\ \mathrm{INV\ DELAY} \\ \mathrm{INV\ CLIN\text{-}M} \end{bmatrix}$$

We can write ($\dot{X}$ denotes the time derivative of $X$):

$$\begin{array}{llllllll}
\dot{X}1=(-r1-\mathrm{d})\times X1 & & & & & & & \mathrm{INS}-\mathrm{SCR1} \\
\dot{X}2=d\times X1 & (-r2-d)\times X2 & & & & & & -\mathrm{SCR2} \\
\dot{X}3= & d\times X2 & (-r3-d)\times X3 & & & & & -\mathrm{SCR3} \\
\dot{X}4= & & d\times X3 & -t2\times X4 & & & & -\mathrm{DIAGSCR} \\
\dot{X}5= & & & & -t2\times X5 & & & L\times Q\times\mathrm{DIAGSCR} \\
\dot{X}6= & & & Q\times t2\times X4 & t2\times X5 & -t3\times X6 & &
\end{array}$$

If we regard the flows INS, SCR1, SCR2, SCR3 and DIAGSCR as inputs, we can write:

$$U=\begin{bmatrix} \mathrm{INS}-\mathrm{SCR1} \\ -\mathrm{SCR2} \\ -\mathrm{SCR3} \\ -\mathrm{DIAGSCR} \\ L\times Q\times\mathrm{DIAGSCR} \\ 0 \end{bmatrix}$$

then we have:

$$\dot{X}=\begin{bmatrix} -r1-d & 0 & 0 & 0 & 0 & 0 \\ d & -r2-d & 0 & 0 & 0 & 0 \\ 0 & d & -r3-d & 0 & 0 & 0 \\ 0 & 0 & d & -t2 & 0 & 0 \\ 0 & 0 & 0 & 0 & -t2 & 0 \\ 0 & 0 & 0 & Q\times t2 & t2 & t3 \end{bmatrix}X+U$$

The input INS is estimated as a function which depends only on the age of a cohort, i.e. INS(age) = tabulated function.

The other inputs, although with negative signs, that affect the model are the flows due to screening. They are:

SCR1(time, cohort) =

$$=S1\times\mathrm{SCRSTAT}\times X1/(S1\times X1+S2\times X2+S3\times X3)$$

SCR2(time, cohort) =

$$=S2\times\mathrm{SCRSTAT}\times X2/(S1\times X1+S2\times X2+S3\times X3)$$

SCR3(time, cohort) =

$$=S3\times\mathrm{SCRSTAT}\times X3/(S1\times X1+S2\times X2+S3\times X3)$$

DIAGSCR (time, cohort) =

$$=Z\times\mathrm{SCRSTAT}\times X4/(S1\times X1+S2\times X2+S3\times X3)$$

It is seen that for any values of S1, S2 and S3 the flows SCR1, SCR2 and SCR3 add up to SCRSTAT, which means that we have built a mechanism for distributing SCRSTAT proportionally to the respective INSITU levels. Our first assumption was that $S1=S2=S3=1$. Other assumptions concerning the distribution of cases detected at screening correspond to other values of the $S1$, $S2$ and $S3$ coefficients and were also tested, but did not give any improvement and the effects on the results were small.

We also assume that $Z=S_{inv}/S_{ins}$ is a constant denoting the ratio of the sensitivity of detecting an INVASIVE case to that of detecting an IN SITU case in the screening process.

DIAGSR is the flow of invasive cases that are detected earlier on account of screening measures. We suppose that the number of such cases is proportional to the number of undiagnosed invasive cases and to the number of screenings.

### Appendix 3. Identification

Identification is a large discipline which includes a number of techniques for different kinds of models, i.e. regression analysis for static models, impulse response, frequency analysis, correlation analysis for non-parametric dynamic models, or least square, maximum likelihood or extended Kalman filtering for parametric dynamical models, and so on. It also discusses choice of model structure, experimental conditions like different ways of perturbing a system, validation techniques, etc. For more information about identification, see e.g. Eykhoff (1974), Box & Jenkins (1970) and Ljung (1987).

In this paper we have used a parametric dynamical model of ordinary differential equation. Input (disturbance) is the elimination of *in situ* cases by screening, and output is the number of invasive diagnoses. Our purpose is to find the model (including a set of parameters whose values are *a priori* unknown) that in a least square sense best fits the system behaviour known from statistical data. This identification technique is often referred to as parameter estimation.

This process is illustrated in Figure 8. The difference in the outputs from the system and the model ($V$) over T years of a study is minimised in a least square sense as:

$$\min V = \frac{1}{T} \cdot \int_0^T e^2(t)\,dt,$$

or, in a discrete form, as:

$$\min V = \frac{1}{T} \cdot \sum_1^T e^2(t)$$

where $e(t)$ is the deviation between model output and corresponding statistics.

Our task was to find the set of parameters which would minimise the loss function $V$. We then performed the identification, first of the prediagnosis and then of the post-diagnosis sub-model.

The first part was based on the course up to the diagnosis of invasive cancer, and the second part was based on the course from diagnosis of invasive cancer to death. In both cases the analyses were made on each of the eight cohorts.

*Identification of the prediagnosis sub-model*

The prediagnosis part of the model (Figure 3) starts with the flow of new *in situ* cases (INS) and of cases eliminated by screening measures (SCR), and ends with diagnoses of invasive cancer (DIAG and DIAGSCR). Our goal was to estimate for each of the eight cohorts in the study the proportion of new *in situ* lesions that progress to invasive cancer ($P$), the time constants for cancer *in situ* (T1) and for preclinical invasive cancer (T2), and the flow of new cancer *in situ* cases (INS). (For further explanation, see Appendix 1 and 2.)

With SCR as a negative input to the system and the model, we wanted to compare the reduction of diagnoses of invasive cases from the system and from the model (Figure 8). We then constructed a loss function ($V1$) which measured the difference between the system and model as a function of $P$, T1, T2 and INS.

$$V1 = \frac{1}{24} \cdot \sum_{1958}^{1981} ((\text{DIAG} + \text{DIAGSCR}) - \text{DIAGSTAT})^2$$

**Figure 8** The identification process for the prediagnosis sub-model – a search for the parameter set which minimises the difference between system (described by available statistics) and model outputs. In the post-diagnosis sub-model the parameters $Q$ and T3 were identified accordingly by minimising $e2(t)$ = MORT – MORTSTAT.

measures this difference in a least square sense. We are then searching for the combination of parameters $P$, T1 and T2 and an INS function that will minimise $V1$. This latter is an optimisation problem, which requires some hundreds of simulation runs per cohort in order to obtain accurate estimates of the parameters and the INS function.

*Identification of the post-diagnosis sub-model*

This part of the system – starting with the diagnosis of invasive cancer and ending with death – could also have been studied by using individual statistics for all patients and calculating the fraction of mortality and the mortality time constant directly. However, it was desirable to perform the whole analysis in a consistent way. Our goal here was to estimate the proportion of diagnosed invasive cases that will die ($Q$) and the time constant from diagnosis to death (T3). However, it was soon found that $Q$ can be regarded as a constant ($Q0$) only up to a certain age ($TQ1$). After this age, an annual increase, $Q1$, has to be added (see Appendix 1). The quantities $Q$ and T3 can be identified both for the case *without* and the case *with* screening.

Technically the estimation of $Q$ and T3 was performed by minimising the loss function $V2$ to get the best least square fit between the system and model. Thus:

$$\min_{Q,T3} V2 = \frac{1}{24} \cdot \sum_{1958}^{1981} (\text{MORT} - \text{MORTSTAT})^2$$

**References**

ALBERT, A. (1981). Estimated cervical cancer disease state incidence and transition rates. *J. Natl Cancer Inst.*, **67**, 572.

ASHLEY, D.J.B. (1966). Evidence for the existence of two forms of cervical carcinoma. *J. Obstet. Gynaecol. Br. Commonw.*, **73**, 382.

BAILAR, J.C. (1961). Uterine cancer in Connecticut. Late deaths among 5-year survivors. *J. Natl Cancer Inst.*, **27**, 239.

BARRON, B.A., CAHILL, M.C. & RICHART, R.M. (1978). A statistical model of the natural history of cervical neoplastic disease: the duration of carcinoma *in situ*. *Gynecol. Oncol.*, **6**, 196.

BIBBO, M., KEEBLER, C.M. & WIED, G.L. (1971). Prevalence and incidence rates of cervical atypia. *J. Reprod. Med.*, **6**, 79.

BIGELOW, J.H. (1975). The natural history of cervical cancer. In *Proceedings of the Joint IIASA/WHO Workshop on Screening for Cervical Cancer*, p. 15. International Institute for Applied Systems Analysis: Laxenburg.

BOURNE, R.G. & GROVE, W.D. (1983). Invasive carcinoma of the cervix in Queensland. *Med. J. Aust.*, **1**, 156.

BOX, G.E.P. & JENKINS, G.M. (1970). *Time Series Analysis Forecasting and Control*. Holden-Day: New York.

BOYES, D.A., FIDLER, K.H. & LOCK, D.R. (1962). Significance of *in situ* carcinoma of the uterine cervix. *Br. J. Cancer*, **16**, 203.

BOYES, D.A., MORRISON, B., KNOX, E.G., DRAPER, G.J. & MILLER, A.B. (1982). A cohort study of cervical cancer screening in British Columbia. *Clin. Invest. Med.*, **5**, 1.

CANADIAN TASK FORCE ON SCREENING (1976). Cervical cancer screening programs. *Can. Med. Assoc. J.*, **114**, 1003.

CANCER INCIDENCE IN SWEDEN. ANNUAL PUBLICATIONS 1958–1981. National Board of Health and Welfare. The Cancer Registry: Stockholm 1960–1984.

CAUSES OF DEATH. ANNUAL PUBLICATIONS 1958–1981. National Central Bureau of Statistics: Stockholm 1960–1983.

CHAMBERLAIN, J. (1984). Failures of the cervical cytology screening programme. *Br. Med. J.*, **289**, 853.

COOK, G.A. & DRAPER, G.J. (1984). Trends in cervical cancer and carcinoma *in situ* in Great Britain. *Br. J. Cancer*, **50**, 367.

COPPLESON, L.W. & BROWN, B. (1974). Estimation of the screening error rate from the observed detection rates in repeated cervical cytology. *Am. J. Obstet. Gynecol.*, **19**, 953.

COPPLESON, L.W. & BROWN, B. (1975). Observations on a model of the biology of carcinoma of the cervix. *Am. J. Obstet. Gynecol.*, **122**, 127.

DRAPER, G.J. & COOK, G.A. (1983). Changing patterns of cervical cancer rates. *Br. Med. J.*, **287**, 510.

DUGUID, H.L.D., DUNCAN, I.D. & CURRIE, J. (1985). Screening for cervical intraepithelial neoplasia in Dundee and Angus 1962–81 and its relation with invasive cervical cancer. *Lancet*, **ii**, 1053.

DUNN, J.E. (1953). The relationship between carcinoma-*in-situ* and invasive cervical carcinoma. *Cancer*, **6**, 873.

DUNN, J.E. & MARTIN, P.L. (1967). Morphogenesis of cervical cancer. Findings from San Diego county cytology registry. *Cancer*, **20**, 1899.

EDDY, D. SHWARTZ, M. (1982). Mathematical models in screening. In *Cancer Epidemiology and Prevention*, Schottenfeld & Fraumeni (eds) p. 1075. Saunders: Philadelphia.

EYKHOFF, P. (1974). *System Identification – Parameter and State Estimation*. John Wiley & Sons: Chichester.

FIDLER, H.K., BOYES, D.A. & WORTH, A.J. (1968). Cervical cancer detection in British Columbia. *J. Obstet. Gynaecol. Br. Commonw.*, **75**, 392.

GUSTAFSSON, L. (1983). Model building and simulation in DYNAMO (in Swedish). UPTEC 8376K, Department of Technology, University of Uppsala.

GUSTAFSSON, L. (1986). The natural history of cancer of the cervix uteri. A simulation study based on Swedish statistics for 1958–1981. Uppsala University Computing Center, UPTEC 8607R, Uppsala.

HABEMA, J.D.F., LUBBE, J.T.N., VAN DER MAAS, P.J. & VAN OORTMARSSEN, G.J. (1983). A computer simulation approach to the evaluation of mass screening. In *Medinfo 1983*, Van Bemmel, J.H., Ball, M.J. & Wigertz, O. (eds) p. 1222. IFIP/IMIA: Amsterdam.

HAKAMA, M. & PENTINNEN, J. (1981). Epidemiological evidence for two components of cervical cancer. *Br. J. Obstet. Gynaecol.*, **88**, 209.

HAKAMA, M., CHAMBERLAIN, J., DAY, N.E., MILLER, A.B. & PROROK, P.C. (1985). Evaluation of screening programmes for gynaecological cancer. *Br. J. Cancer*, **52**, 669.

IARC WORKING GROUP ON EVALUATION OF CERVICAL CANCER SCREENING PROGRAMMES (1986). Screening for squamous cervical cancer: duration of low risk after negative results of cervical cytology and its implication for screening policies. *Br. Med. J.*, **293**, 659.

IMSL LIBRARY (1982). *Volumes 1–4*, 9th edn.

KASHGARIAN, M. & DUNN, J.E. (1970). The duration of intraepithelial and preclinical squamous cell carcinoma of the uterine cervix. *Am. J. Epidemiol.*, **92**, 783.

KASPER, T.A., SMITH, E.S.O., COOPER, P., CLAYTON, J. & TODD, D. (1970). An analysis of the prevalence and incidence of gynecologic cancer cytologically detected in a population of 175,767 women. *Acta Cytol.*, **14**, 261.

KESSLER, I.I. (1974). Perspectives on the epidemiology of cervical cancer with special reference to the herpesvirus hypothesis. *Cancer Res.*, **34**, 1091.

KNOX, E.G. (1975). Computer simulation studies of alternative population screening policies. In *Systems Aspects of Health Planning*, Bailey, N.T.J. Thompson, M. (eds). North-Holland: Amsterdam.

KNOX, E.G. (1982). Cancer of the uterine cervix. In *Trends in Cancer Incidence. Causes and Practical Implications*, Magnus, K. (ed) p. 271 McGraw-Hill: New York.

KOSS, L.G. (1979). *Diagnostic cytology and its histopathologic bases*, p. 285 Lippincott: Philadelphia.

LJUNG, L. (1987). *System Identification: Theory for the User*. Prentice Hall: Englewood Cliffs, NJ.

LÄÄRÄ, E., DAY, N.E. & HAKAMA, M. (1987). Trends in mortality from cervical cancer in the nordic countries: association with organised screening programmes. *Lancet*, **i**, 1247.

MATTSSON, B., & WALLGREN, A. (1984). Completeness of the Swedish Cancer Register. Non-notified cancer cases recorded on death certificates in 1978. *Acta Radiol. Oncol.*, **23**, 305.

MILLER, A.B. (1982). The Canadian experience of cervical cancer: incidence trends and a planned natural history investigation. In *Trends in Cancer Incidence. Causes and Practical Implications*, Magnus, K. (ed) p. 311. McGraw-Hill: New York.

NATIONAL BOARD OF HEALTH AND WELFARE (1982). Principles and routines for gynecologic health examinations. Report from group of experts of National Board of Health and Welfare (in Swedish). Stockholm.

NATIONAL CENTRAL BUREAU OF STATISTICS. Population December 31 1957–1981. Stockholm. Annual publications 1959–1983.

PARKIN, D.M., HODGSON, P. & CLAYDEN, A.D. (1982). Incidence and prevalence of preclinical carcinoma of cervix in a British population. *Br. J. Obstet. Gynaecol.*, **89**, 564.

PARKIN, D.M. (1985). A computer simulation model for the practical planning of cervical cancer screening programmes. *Br. J. Cancer*, **51**, 551.

PARKIN, D.M. & MOSS, S.M. (1986). An evaluation of screening policies for cervical cancer in England and Wales using a computer simulation model. *J. Epidemiol. Comm. Hlth*, **40**, 143.

PATERSON, M.E.L., PEEL, K.R. & JOSLIN, C.A.F. (1984). Cervical smear histories of 500 women with invasive cervical cancer in Yorkshire. *Br. Med. J.*, **289**, 896.

PETERSEN, O. (1956). Spontaneous course of cervical precancerous conditions. *Am. J. Obstet. Gynecol.*, **72**, 1063.

PETTERSSON, F., BJÖRKHOLM, E. & NÄSLUND, I. (1985). Evaluation of screening for cervical cancer in Sweden: trends in incidence and mortality 1958–1980. *Int. J. Epidemiol.*, **14**, 521.

PUGH, A.L., III (1976). *DYNAMO User's Manual*. MIT Press: Cambridge, MA.

ROBRA, B.-P., SCHWARTZ, F.W. & BRECHT, J.G. (1985). Evaluation of the screening program for cervical cancer in the Federal Republic of Germany from an epidemiological perspective. In *Cancer Campaign, vol. 8, Cancer of the Uterine Cervix*, Grundmann, E. (ed) p. 23 Fischer Verlag: Stuttgart, New York.

THORN, J.B., RUSSELL, E.M., MACGREGOR, J.E. *et al.* (1975). Costs of detecting and treating cancer of the uterine cervix in north-east Scotland in 1971. *Lancet*, **i**, 674.

WAIT, J.W. & CLARK, D. (1977). *DARE-P User's Manual, version 4.1*. University of Arizona, College of Engineering, Arizona.

YU SHUN-ZHANG, MILLER, A.B. & SHERMAN, G.J. (1982). Optimising the age, number of tests and test interval for cervical cancer screening in Canada. *J. Epidemiol. Comm. Hlth*, **36**, 1.